# Effect of a High-Intensity Dietary Intervention on Changes in Dietary Intake and Eating Pathology during a Multicomponent Adolescent Obesity Intervention

**DOI:** 10.3390/nu13061850

**Published:** 2021-05-28

**Authors:** Hollie A. Raynor, Suzanne E. Mazzeo, Jessica Gokee LaRose, Elizabeth L. Adams, Laura M. Thornton, Laura J. Caccavale, Melanie K. Bean

**Affiliations:** 1 Department of Nutrition, University of Tennessee Knoxville, 1215 W. Cumberland Ave., Knoxville, TN 37996, USA; hraynor@utk.edu; 2 Department of Pediatrics, School of Medicine, Children’s Hospital of Richmond at Virginia Commonwealth University, Box 980140, Richmond, VA 23298, USA; semazzeo@vcu.edu (S.E.M.); Elizabeth.Adams@vcuhealth.org (E.L.A.); Laura.Caccavale@vcuhealth.org (L.J.C.); 3 Department of Psychology, College of Humanities and Sciences, Virginia Commonwealth University, Box 842018, Richmond, VA 23284, USA; 4 Department of Health Behavior and Policy, School of Medicine, Virginia Commonwealth University, Box 980430, Richmond, VA 23298, USA; Jessica.larose@vcuhealth.org; 5 Department of Psychiatry, University of North Carolina at Chapel Hill, 101 Manning Drive, Chapel Hill, NC 27599, USA; laura_thornton@med.unc.edu; 6 Department of Psychiatry, School of Medicine, Virginia Commonwealth University, Box 980308, Richmond, VA 23298, USA

**Keywords:** adolescent, energy intake, diet quality, eating pathology, obesity treatment

## Abstract

Concerns remain about dietary changes during pediatric obesity treatment and eating pathology, which have not been investigated. This secondary data analysis from a randomized clinical trial examined associations between adolescents’ changes in energy intake and diet quality during obesity treatment with post-treatment eating pathology. Adolescents (N = 82: 13.7 ± 1.2 y, 34.9 ± 7.0 kg/m^2^, 63.4% female, 46.3% black) received TEENS+, a 4-month multicomponent intervention. TEENS+ provided individualized dietary goals (1200–1800 kcal/day; number of “Go” foods/day (low-energy, high-nutrient-dense foods)). At 0 and 4 months, 3-day food records assessed energy intake and diet quality (Healthy Eating Index 2015 (HEI-2015)). Two HEI-2015 subscores were created: components to increase (increase), and components to limit (decrease). The Eating Disorder Examination Questionnaire measured eating pathology (total score and subscales: restraint; and eating, weight, and shape concern). Corrected *p*-values are reported as q-values. Energy intake decreased (−292 ± 418 kcal/day; q < 0.001), while diet quality improved during treatment (total HEI-2015 (4.5 ± 15.1; q = 0.034) and increase (3.3 ± 9.4; q = 0.011)). Restraint increased (+0.6 ± 1.4; q < 0.001), whereas shape (−0.5 ± 1.3; q = 0.004) and weight (−0.5 ± 1.4; q = 0.015) concerns decreased. Greater decreases in energy intake were associated with greater restraint post-treatment (F = 17.69; q < 0.001). No other significant associations were observed. Changes in adolescents’ dietary intake during obesity treatment were unrelated to increased shape, weight, or eating concerns post-treatment.

## 1. Introduction

Graded recommendations for the treatment of overweight and obesity during adolescence have been developed, which encourage starting with a multicomponent intervention [1,2]. This intervention includes diet and physical activity and/or sedentary behavior goals combined with a family-based behavioral intervention to assist with changing energy balance behaviors. Although the multicomponent intervention has a graded recommendation [1,2], there are no specific dietary interventions that have graded recommendations to be used within this approach. Thus, at this time, dietary interventions recommended for pediatric overweight and obesity treatment are fairly broad [3], ranging from only changing specific parts of the diet, such as food group intake (e.g., decreasing sugar-sweetened beverages (SSBs), increasing fruits and vegetables) to changing the overall diet (e.g., the Traffic Light Diet [4], which includes daily energy goals and daily servings from food groups that correspond to the colors of the traffic light). Dietary interventions that change only certain parts of the diet are considered to provide less structure to the diet and could be considered to be lower intensity, potentially having less impact on overall dietary intake [5]. Dietary interventions that change many parts of the diet provide more structure in the diet and could be considered to be higher intensity, as they have the ability to alter overall dietary intake [5]. Although not empirically tested with children or adolescents, dietary interventions that provide more structure in the diet during treatment of overweight and obesity with adults have yielded greater weight loss compared to dietary interventions providing less structure [6]. 

Although a high-intensity dietary intervention might produce greater changes in dietary intake and enhance weight loss outcomes, there are concerns that this type of dietary intervention, particularly one that focuses on reducing intake of specific nutrients (i.e., energy) and/or types of foods, might promote increases in eating pathology in adolescents, a population at higher risk for the development of eating disorders [7]. This concern has arisen due to the association between self-reported dieting and eating pathology in observational research [8]. For example, in children aged nine to 14 years (N = 16,882) who were followed for two years, self-reported dieting was associated with increased binge eating [9]. Similarly, in a longitudinal study in which 2516 adolescents were followed for five years, self-reported dieting behaviors were associated with increased risk of binge eating at the 5-year follow-up [10]. Moreover, among 888 adolescents who were followed for three years, self-reported dieting was related to a five- to 18-fold increased risk of developing an eating disorder [11]. In this latter study, participants who self-reported severely restricting energy intake and skipping meals were those at greatest risk for the development of an eating disorder [11]. As such, dietary interventions which emphasize reducing overall dietary intake might be the most problematic in regard to the development of eating pathology. However, investigations with more ability to determine causal relationships between reducing overall dietary intake and eating pathology during adolescence have not been conducted.

Whereas high-intensity dietary interventions for the treatment of overweight and obesity in adolescents commonly focus on reducing intake of energy and/or non-nutrient-dense, energy-dense foods, these interventions may also focus on increasing intake of nutrient-dense, low-energy-dense foods (i.e., fruits and vegetables) to enhance the quality of the diet and to assist with appetite regulation [12]. As it is surmised that self-reported dieting captured in previous research represents restricting intake [13], it is not clear at this time if a dietary intervention that includes prescribed reduced-energy goals and also focuses on increasing intake of certain foods is associated with increased risk of eating pathology in adolescents during treatment of overweight and obesity. Moreover, if there is a relationship between changes in dietary intake and eating pathology, it is not known if this relationship occurs between all changes in dietary intake or only changes related to reducing intake.

Two systematic reviews have recently examined if eating pathology and/or eating disorder risk increases in children and adolescents with overweight or obesity receiving a multicomponent intervention for treatment [8,14]. Both reviews reported that the included investigations found no increase or a reduction in eating pathology or eating disorder risk [8,14]. One review reported that dietary restraint or dieting increased, but eating pathology and eating disorder risk did not [14]. Although these reviews included investigations with dietary interventions, the actual association between changes in the diet and eating pathology were not reported, and to the authors’ knowledge, this relationship has not been examined in any previous study. To address this gap, the purpose of this secondary data analysis was to quantify pre- to post-treatment changes in adolescents’ daily energy intake, dietary quality, and eating pathology during a 4-month multicomponent intervention that included a high-intensity dietary intervention for the treatment of overweight and obesity (TEENS+) [15]. Further, the relationship between changes in energy intake and diet quality during treatment with post-treatment eating pathology was examined. As the TEENS+ dietary intervention provided goals to decrease energy intake and increase intake of nutrient-dense, low-energy-dense foods, it was hypothesized that there would be significant decreases in daily energy intake, significant improvement in dietary quality due to increases in consumption of healthier food groups (i.e., fruits and vegetables), and significant decreases in consumption of less healthy food groups (i.e., foods high in added sugars). It was also hypothesized that there would be no significant change in eating pathology from pre- to post-treatment, and that reductions in daily energy intake would be related to increased restraint, but that other changes in the diet would not be associated with other measures of eating pathology.

## 2. Materials and Methods

### 2.1. Study Design and Participants

This secondary data analysis was drawn from a randomized clinical trial, conducted in 2016–2018 in Virginia, in which adolescents and one adult caregiver were randomized into one of two 4-month interventions: TEENS + PAC (parents as coaches) or TEENS + PWL (parent weight loss). For this investigation, data collected at the pre- and post-treatment assessments were used. This trial was registered in ClinicalTrials.gov (NCT#02586090) and was approved by the Institutional Review Board of Virginia Commonwealth University.

Participants (adolescents and adult caregivers) were recruited primarily through pediatrician referrals and direct mailings. Eligible adolescents were aged 12–16 years, with a body mass index (BMI) >85th percentile for age and sex [16] and who primarily resided in the participating caregiver’s home. Exclusion criteria included: (1) a clinical eating disorder (anorexia nervosa, bulimia nervosa, or recent compensatory behavior) or significant psychopathology (e.g., suicidality, psychosis); (2) inability to follow the protocol due to a physical or developmental disorder (e.g., mobility or severe autism); (3) change in medications that could impact weight (e.g., antidepressants) or use of atypical antipsychotics or steroids within the past 3 months; (4) diabetes mellitus; and (5) medical conditions known to impact weight or eating (e.g., Prader Willi Syndrome, history of bariatric surgery). Adult caregivers were >18 years of age with BMI > 25 kg/m^2^ with the same exclusion criteria as those applied for adolescents; however, adults with type 2 diabetes were eligible if they were on a >3-month stable dose of diabetes medications. All participants were required to receive medical clearance prior to participation. For these secondary data analyses, only data collected from the adolescents were examined.

### 2.2. Interventions

#### 2.2.1. TEENS+ Adolescent Intervention

All adolescents received the TEENS+ intervention. The intervention was delivered in weekly group meetings, in which adolescents attended same-sex groups led by two behavior coaches (psychology doctoral trainees or similar and registered dietitians). These weekly group meetings included 1-h group visits in which the content of the intervention was delivered, and 1-h supervised exercise sessions. Adolescents also received one 30-min individual visit per month (four total individual sessions over the four-month intervention). Two of these meetings were with their behavior coach and designed to support motivation for change, using a motivation-interviewing (MI)-informed approach, and two were designed to assist with dietary modifications and were with a study dietitian.

Sessions were implemented using a manualized protocol, which included a behavior therapy approach. Participants were taught skills, including guided goal-setting and self-monitoring, identifying barriers and solutions, contingency management, stimulus control, setbacks, and relapse prevention, to assist with changing behaviors. Session content also included strategies to attend to hunger and satiety cues; regulate eating patterns; and effectively manage social, emotional, and environmental cues while remaining adherent to healthy lifestyle goals. At each session, weight was measured during an individual check-in with the behavior coaches. Adolescents were encouraged to begin self-weighing once per week on the morning of group during the final month of treatment (weeks 13–16) to assist in application of the self-regulation process and prepare them for the maintenance phase. Participants self-monitored daily dietary intake and physical activity, with coaches providing weekly personalized feedback, using a self-regulation framework.

The dietary intervention was designed to reduce energy intake and increase intake of low-energy, nutrient-dense foods (“Go Foods”), such as fruits and vegetables. Participants were given an individualized daily energy goal that was based upon sex and height, and ranged from 1200–1400 kcal for females and 1500–1800 kcal for males. An individualized “Go Food” goal, based on baseline dietary intake of “Go Foods,” was also provided. These dietary goals were designed to assist with achieving a 1–2 lb/week weight loss. “Go Food” goals were increased as previous goals were met, and energy goals were adjusted as needed throughout treatment based on the rate of weight change. Standardized nutrition education lessons provided information about energy balance, macronutrients, and eating behaviors associated with obesity (e.g., sugared beverage intake and meals away from home).

The TEENS+ exercise goals were to achieve ≥1 h/day of moderate- to vigorous-intensity physical activity. Exercise occurred at the program gym, YMCA (caregiver and adolescent memberships provided), and other locations. Adolescents exercised >1 x/week at the program gym during the weekly sessions.

#### 2.2.2. Adult Caregiver Interventions

Adult caregivers attended weekly group sessions for the intervention for which they had been assigned (PAC or PWL). These interventions were matched on contact time, and led by trained, supervised behavior coaches.

Parents as Coaches (PAC): PAC focused on skills training based on authoritative parenting and feeding strategies to support adolescents’ weight management. PAC emphasized parent role modeling and developing a home environment that supported healthy eating and activity behaviors. This intervention did not focus on adult weight and did not monitor adult weight. During the four-month intervention, there were 8 parent skills training sessions (parents attended alone) and 8 nutrition education sessions (parents attended with adolescents). Adults self-monitored key parenting behaviors and received personalized written feedback that taught them to identify connections between their behaviors and their adolescents’ weekly weight change.

Parent Weight Loss (PWL): In PWL, adults were provided with personalized energy, fat, and physical activity goals to achieve a weight loss goal of 1–2 lbs/week. They also self-monitored key information (e.g., weight, dietary intake, and exercise) on a daily basis and were taught to use these data to guide their choices within a self-regulation framework to assist with weight change. PWL emphasized consuming “Go Foods” to meet energy goals, enhance satiety and diet quality. Parents were trained in behavioral weight loss strategies (e.g., goal setting, stimulus control) and techniques to help them meet their own diet, physical activity, and weight loss goals [17]. Their weight was measured weekly, and they received tailored feedback and coaching to assist them in their weight loss efforts within the context of their personal values and environmental context. PWL did not focus on adolescents’ weight management directly.

### 2.3. Measures

Assessments were conducted by masked assessors at 0 (pre-treatment) and 4 months (post-treatment). Families received $40 at the 4-month assessment. Surveys were completed online using REDCap.

#### 2.3.1. Sociodemographics

At baseline, caregivers reported on adolescent age, sex, race, and ethnicity; household income; and insurance status.

#### 2.3.2. Anthropometrics

Adolescent height and weight were measured after a 12-h fast in light clothing to the nearest 0.1 cm and 0.1 kg using a wall-mounted stadiometer (Seca 213) and electronic digital scale (Scale-Tronix 5125-X), respectively. The average of three measures was used. Adolescent BMI (kg/m^2^) and adolescent BMI percentile were calculated using Epi Info Software [18].

#### 2.3.3. Dietary Intake

Adolescents completed 3-day food records (parent-assisted as needed), which included one weekend day and two weekdays. Food logs were reviewed with a study dietitian, using food models and portion guides to obtain additional details (e.g., portion sizes, cooking methods, brands). Data were entered into Nutrition Data Systems for Research (NDSR; Nutrition Coordinating Center, University of Minnesota, Minneapolis, Minnesota). Total energy intake (average of the three days) was calculated. Dietary quality was determined using the Healthy Eating Index 2015 (HEI-2015) [19]. The HEI-2015 is comprised of 13 scored components: total fruits, whole fruits, total vegetables, greens and beans, whole grains, dairy, total protein foods, seafood and plant proteins, fatty acids, refined grains, sodium, added sugars, and saturated fats. Scores range from 0 to 100 and are calculated based upon densities (i.e., the amount of a dietary component per 1000 kcal). Higher scores are reflective of higher dietary quality. Along with the total score, two composite subscores were developed. One subscore, “Increase,” was comprised of components of the HEI-2015 that are recommended to increase, with greater amounts consumed per density receiving higher scores (total fruits, whole fruits, total vegetables, greens and beans, whole grains, dairy, total protein foods, seafood and plant proteins, and fatty acids). Possible scores for the increase subscore ranged 0 to 60. The second subscore, “Decrease,” contained components of the HEI-2015 that are recommended to limit (eat in moderation), with lower amounts consumed per density receiving higher scores (refined grains, sodium, added sugars, and saturated fats). Possible scores for the decrease subscore ranged 0 to 40.

#### 2.3.4. Eating Pathology

Adolescents completed the 36-item Eating Disorder Examination Questionnaire (EDE-Q), a widely used measure of eating disorder symptomatology with demonstrated validity and reliability among adolescents [20]. The EDE-Q measures eating disorder features over the previous 28 days. Although there are 36 items, scores on each of four subscales, as well as the total score, may be derived from 22 items. The EDE-Q has four subscales: restraint (5 items), eating concern (5 items), weight concern (5 items), and shape concern (8 items), and an overall score (mean of the four subscales). Scores range from 0 to 6, with higher scores on the subscales and on the total score indicating greater eating pathology.

### 2.4. Statistical Analysis

All adolescents received the same intervention, and although there was a significant reduction in BMI, there were no significant differences in adolescent BMI reductions at 4 months between the two interventions to which the families had been randomized (TEENS + PAC or TEENS + PWL) [15]. Thus, data for adolescents were combined into one group for the analyses, with models controlling for randomized intervention. Participant characteristics were summarized using frequencies, percentages, and means and standard deviations (SD). To quantify changes in adolescents’ daily energy intake, dietary quality, and eating pathology, repeated measures analysis of variance models using PROC MIXED were used to examine changes in daily energy intake (kcal/day), dietary quality (HEI total score and the two HEI composite subscores), and eating pathology (EDE-Q total score; EDE-Q subscales) from pre- to post-treatment. All models included adolescent sex and randomized intervention as covariates. To examine the relationship between changes in energy intake and diet quality during treatment with post-treatment eating pathology, change scores for energy intake and dietary quality from pre- to post-treatment were calculated as post-treatment minus pre-treatment; thus, negative scores indicated a decrease, and positive scores indicated an increase, in these values. Linear models using PROC MIXED were then used to examine if the independent variables of change score values for energy intake and dietary quality were associated with the dependent variables of eating pathology (EDE-Q total score; EDE-Q subscale scores) post-treatment. All models controlled for EDE-Q pre-treatment values, adolescent sex, and randomized intervention. When reporting model statistics related to changes in kcal/day, the beta value and standard error were multiplied by 100 for ease of interpretability (e.g., beta values per 100 kcal, rather than per 1 kcal).

For all models, intent-to-treat analyses were applied by carrying pre-treatment values forward for any missing values at post-treatment. False Discovery Rate was applied to correct for multiple testing, where corrected *p*-values (indicated as q-values) <0.05 were considered significant [21]. Analyses were conducted using SAS Studio version 3.8.

## 3. Results

Eighty-two adolescents were randomized into the trial (see Table 1 for participant characteristics), with 85% retained at 4 months. There were no differences in 4-month retention in adolescent demographics (ps > 0.05), but adolescents not retained at 4-months had a greater pre-treatment BMI compared to those who were retained (*p* = 0.046). Adolescents’ daily energy intake, dietary quality, and eating pathology at pre- and post-treatment are shown in Table 2. Daily energy intake significantly decreased during treatment by an average of −292 ± 418 kcal/day (range: −1828 to +539 kcal/day; q < 0.001). Diet quality significantly improved, as indicated by an increase in overall HEI scores (4.5 ± 15.1 units; range: −35.5 to 52.8; q = 0.034). Further examination of HEI subscores indicated that the increase subscore significantly improved by 3.3 ± 9.4 units (range: −22.0 to 36.8; q = 0.011); however, there was no significant change in the decrease subscore (1.2 ± 6.9; range: −16.7 to 22.4; q = 0.430) over time. Adolescents’ eating pathology showed a significant increase in restraint from pre- to post-treatment (+0.6 ± 1.4 units; range: −2.6 to 6.0; q < 0.001), with a significant decrease in concern about shape (−0.5 ± 1.3 units; range: −4.9 to 2.4; q = 0.004) and weight (−0.5 ± 1.4 units; range: −4.3 to 3.5; q = 0.015). Adolescents’ concern about eating and total EDE-Q scores did not significantly change from pre- to post-treatment (concern about eating: 0.04 ± 1.1 units; range: −4.2 to 2.6; total EDE-Q scores: −0.1 ± 1.0 units; range: −3.9 to 2.2; qs > 0.05).

A greater decrease in daily energy intake from pre- to post-treatment was associated with greater restrained eating at post-treatment (F-value [q-value] = 17.7 [q < 0.001]). The beta value was −0.1, indicating that a 100 kcal/day greater decrease in daily energy intake from pre- to post-treatment was associated with a 0.1 unit increase in restraint subscale scores. There were no significant associations between changes in daily energy intake and other indicators of eating pathology, including EDE-Q total scores, and concern about shape, weight, and eating at post-treatment (qs > 0.05). Further, there were no significant associations between changes in dietary quality across treatment (HEI total score, increase subscore, decrease subscore) on EDE-Q total or subscale scores at post-treatment (qs > 0.05).

## 4. Discussion

Multicomponent interventions for the treatment of overweight or obesity in children and adolescents typically provide dietary goals to assist with reducing energy intake to improve weight status [1]. Although previous research has found no relationship between multicomponent treatments and increased eating disorder risk [8,14], there are still concerns that dietary interventions, particularly those that include goals to reduce energy intake, may be problematic [22]. This concern is driven by the relationship between self-reported dieting and eating pathology in observational research [7]. Thus, this investigation examined changes in the diet that occur when a high-intensity dietary intervention—one that provides explicit goals to reduce energy intake and increase intake of low-energy, high-nutrient foods—is implemented during a multicomponent obesity treatment. This study also examined how dietary changes, both those that show a reduction (i.e., energy) and an increase (i.e., low-energy, high-nutrient foods) in intake, are related to eating pathology in adolescents with overweight or obesity.

In this investigation, the observed changes in dietary intake reflected the explicit goals that were provided in the treatment. Energy intake significantly decreased, by approximately 300 kcal per day, with the mean reported post-treatment daily energy intake in the mid-range of the provided energy goals. The HEI-2015 total score also increased, showing improvement in diet quality, with the subscores from the HEI-2015 indicating that the increase in diet quality was due to greater intake of, or a greater proportion of the diet occurring from, components of the diet that are recommended to increase (HEI-2015 increase subscore). This suggests that there was an increase in intake of “Go Foods,” or at least a greater proportion of the diet was “Go Foods,” which was an intervention dietary goal. Although increasing intake of low-energy, nutrient-dense foods, such as “Go Foods,” might be hypothesized to assist with reducing intake of high-energy, non-nutrient-dense foods, previous pediatric research has shown that there is not an automatic substitution of these foods [23]. This lack of automatic substitution, combined with the fact that the intervention did not have explicit goals to reduce intake of high-energy, non-nutrient-dense foods in the dietary intervention, might help explain why there was no increase in the HEI-2015 decrease subscore (indicating decreased intake or a smaller proportion of the diet consumed was from these types of foods). Importantly, the 2015–2016 National Health and Examination Survey found that the average total HEI-2015 score for children aged 12 to 17 years was 51.8 (similar to what was seen at baseline). Thus, the TEENS+ dietary intervention was successful at enhancing dietary quality, compared to typical intake for this age group [24].

While the changes in dietary intake reflected the treatment goals, it is important to note that there was a large amount of variability in the response. This amount of variability to intervention response has been one of the major reasons why the focus on “precision medicine” has developed [25]. A component of this is precision nutrition, which is focused on identifying moderators to dietary treatment response. It is believed that identifying response phenotypes will allow the field to further personalize dietary interventions to optimize outcomes.

The majority of the scores from the EDE-Q showed no significant change or significant decrease, post-treatment. Only one subscale, restraint, showed a significant increase post-treatment. These results are similar to two systematic reviews in which eating pathology and/or eating disorder risk were examined in children and adolescents with overweight or obesity receiving a multicomponent intervention for treatment [8,14]. The current investigation adds to this literature by specifically examining the relationship between changes in the diet, rather than just participation in treatment, and eating pathology. Collectively, findings suggest that supervised dietary interventions within obesity treatment are not associated with increased eating pathology, which is in contrast to the observed relationships between unsupervised “dieting” and increased eating pathology among adolescents with obesity [25]. In this investigation, mean EDE-Q scores, both pre- and post-treatment, are well below the clinical cut-off score of >4 [26]. Compared with EDE-Q scores from white, female adolescents aged 12 to 14 years of varying weight status [26], or black, undergraduate women of varying weight status [27], the EDE-Q scores reported in this investigation were slightly higher. As BMI has been positively related to EDE-Q scores in adult women, the slightly higher EDE-Q scores found in this investigation may reflect the sample’s elevated BMI, in contrast to the prior studies that included participants with BMIs across the weight spectrum [28]. 

As noted previously, the restraint subscale on the EDE-Q did increase, and reductions in energy intake were related to restraint at post-treatment. Although increased intake (or a greater proportion of the diet) of low-energy, nutrient-dense foods occurred, this dietary change was not related to post-treatment restraint. Thus, the relationship between decreases in energy intake and post-treatment restraint most likely occurred due to adolescents’ endorsement of behaviors commonly used to successfully reduce energy intake (i.e., limit the amount of food to eat, excluding liked foods, following rules regarding eating (e.g., a calorie limit)). Thus, in this investigation, increases in restraint might be measuring actual success at dietary change regarding energy intake. Restraint increased and was related to decreased energy intake, yet eating, shape, and weight concerns decreased or stayed the same, suggesting that the adolescents may have been able to implement a “healthy” type of restraint or a “health-promoting” restraint [7]. Schaumberg and colleagues propose that the “health-promoting” restraint can occur when a successful self-regulation process is implemented [7]. This process includes three components: self-monitoring, self-evaluation (comparing self-monitoring data to goals), and self-reinforcement (reacting to self-evaluation) [7]. Indeed, multicomponent interventions for the treatment of overweight or obesity, including the intervention in the current investigation, implement this self-regulation framework. Alternatively, restraint may become problematic and increase risk for eating pathology when the self-regulation process breaks down [7]. This breakdown may be more likely when “dieting” occurs outside the context of a multicomponent program. To better understand this relationship, future research should examine restraint, energy intake and energy balance state (weight loss, gain, and maintenance), and the consistency of how self-regulation is implemented.

Strengths of the investigation included a diverse sample, valid measures of dietary intake and eating pathology, and use of the HEI-2015 to establish diet quality. Limitations included self-reported dietary intake, a study design that limited the ability to infer causation, and the relatively brief length of the intervention. Future investigations should examine multicomponent interventions for the treatment of adolescent overweight and obesity with differing levels of dietary structure (i.e., low-intensity vs. high-intensity diets) that are implemented over longer periods of time, such as 12 months, to assist with determining the effect of the intensity of the dietary intervention on the long-term development of eating pathology. Additionally, the course of eating pathology or eating disorder risk should be examined longitudinally as adolescents transition to young adults and when obesity treatment support has been removed.

## 5. Conclusions

During a 4-month multicomponent intervention for the treatment of adolescent overweight and obesity, a high-intensity dietary intervention reduced energy intake and enhanced diet quality. Furthermore, decreases in energy intake were related to higher post-treatment restraint. Eating, shape, and weight concerns did not increase post-treatment, suggesting that increases in restraint during a multicomponent intervention that are associated with a high-intensity diet may not increase risk of eating pathology in the short-term. Longer-term follow up is needed to examine the course of eating pathology once treatment ends and as this population transitions from adolescence into emerging adulthood.

## Figures and Tables

**Table 1:** Baseline demographics of adolescents (N = 82) enrolled in TEENS+.

Female, n (%)	52 (63.4)
Race ^a^, n (%)	
African American	38 (46.3)
White	41 (50.0)
Asian	3 (3.7)
Native American	2 (2.4)
Other	5 (6.1)
Hispanic, n (%)	4 (4.9)
Family insurance status ^b^, n (%)	
None	2 (2.5)
Medicaid	17 (21.3)
Private Insurance	61 (76.3)
Annual family income ^b^, n (%)	
<$10,000	2 (2.5)
$10,000–$20,000	7 (8.8)
$20,000–$30,000	8 (10.0)
$30,000–$40,000	6 (7.5)
$40,000–$50,000	2 (2.5)
$50,000–$75,000	23 (28.8)
$75,000–$100,000	13 (16.3)
$100,000–$150,000	16 (20.0)
>$150,000	3 (3.8)
Age (yrs), mean (SD)	13.7 (1.2)
Weight (kg), mean (SD)	94.3 (19.4)
BMI (kg/m^2^), mean (SD)	34.9 (7.0)
BMI percentile, mean (SD)	98.4 (1.4)

^a^ n = 2 adolescents declined to provide race information; participants could select more than 1 racial category; thus, percentages do not total 100%. ^b^ Parent-reported at baseline. SD = standard deviation; BMI = body mass index.

**Table 2:** Daily energy intake, dietary quality, and eating pathology values at pre- and post-treatment, following a 4-month multicomponent obesity intervention in N = 82 adolescents. Test statistics are from repeated measures analysis of variance models examining changes from pre- to post-treatment.

	Pre-Treatment Mean (SD)	Post-Treatment ^a^ Mean (SD)	F-Value (q-Value ^b^)
Dietary intake and quality			
Energy (kcal/day)	1765 (508)	1473 (461)	39.9 (<0.001)
HEI overall score ^c^	51.0 (12.8)	55.5 (13.2)	7.3 (0.034)
HEI increase subscore ^d^	31.0 (8.5)	34.4 (8.8)	10.3 (0.011)
HEI decrease subscore ^e^	19.9 (5.4)	21.2 (5.6)	2.5 (0.430)
Eating Disorders Examination Questionnaire (EDE-Q) ^f^			
Total score	2.1 (1.1)	2.0 (1.0)	0.5 (0.860)
Restraint subscale	1.3 (1.2)	1.9 (1.3)	17.1 (<0.001)
Eating concern subscale	1.3 (1.1)	1.3 (1.2)	0.1 (0.959)
Shape concern subscale	2.8 (1.6)	2.3 (1.5)	12.9 (0.004)
Weight concern subscale	2.9 (1.4)	2.4 (1.2)	9.3 (0.015)

^a^ Post-treatment was at 4-month time point. ^b^ q-values are corrected *p*-values using false discovery rate for multiple testing. ^c^ Possible range = 0–100, with higher scores indicating better dietary quality. ^d^ Possible range = 0–60, with higher scores indicating better dietary quality. ^e^ Possible range = 0–40, with higher scores indicating better dietary quality. ^f^ Possible range = 0–6, with higher scores indicating greater eating pathology. SD = standard deviation. HEI = healthy eating index—2015.

## Data Availability

Following the publication of associated research findings, M.K.B. will accept data-sharing requests from qualified investigators within the greater scientific community. All requests for data sharing will be reviewed and approved by the M.K.B. prior to the release of data. Shared datasets and corresponding data dictionaries would be free of identifiers or variables that would permit linkage to or lead to deductive disclosure of the identity of individual subjects. All data-sharing procedures would be in compliance with institutional and IRB policy at Virginia Commonwealth University, NIH policy, HIPAA, and other local, state, and federal laws and regulations.
